# Associations between erythropoietin polymorphisms and risk of diabetic microvascular complications

**DOI:** 10.18632/oncotarget.22699

**Published:** 2017-11-27

**Authors:** Hua Li, Huipu Xu, Yuerong Li, Dongdong Zhao, Baoxin Ma

**Affiliations:** ^1^ Department of Oncology, The Affiliated Hospital of Binzhou Medical University, Binzhou, Shandong 256603, China; ^2^ Department of Cardiology, The Affiliated Hospital of Binzhou Medical University, Binzhou, Shandong 256603, China

**Keywords:** erythropoietin, polymorphism, diabetic microvascular complication, systematic review, meta-analysis

## Abstract

We conducted a meta-analysis to evaluate the relationship between erythropoietin (EPO) polymorphisms and diabetic microvascular complications. We searched the PubMed, Embase, Cochrane library, Web of Science, Wanfang, and Chinese National Knowledge Infrastructure databases for appropriate studies. Odds ratios (ORs) with 95% confidence intervals (CIs) were calculated to evaluate the associations. Ultimately, eight studies consisting of 2,861 cases and 2,136 controls were identified and included in our meta-analysis. Results with our genotype model indicated an association between rs1617640 polymorphisms and diabetic microvascular complications (TT vs. GG: OR = 1.544, 95% CI = 1.089–2.189, *P* = 0.015). No clear associations between the rs1617640 and rs507392 polymorphisms and diabetic retinopathy were observed. By contrast, rs551238 polymorphisms were associated with increased diabetic retinopathy risk (allele model: OR = 0.774, 95% CI = 0.658–0.911, *P* = 0.002; genotype model: AC vs. CC: OR = 0.598, 95% CI = 0.402–0.890, *P* = 0.011; dominant model: OR = 0.561, 95% CI = 0.385–0.817, *P* = 0.003; recessive model: OR = 0.791, 95% CI = 0.643–0.973, *P* = 0.026). These results indicate that EPO polymorphisms are a risk factor for diabetic microvascular complications.

## INTRODUCTION

Diabetes prevalence has increased globally from 4.7% in 1980 to 8.5% in 2014 among adults over 18 years of age [1]. Diabetes can damage blood vessels, eyes, and kidneys, resulting in microvascular complications [1–3], and is a contributing factor in 2.6% of global blindness cases [1, 4].

Erythropoietin (EPO) is a kidney-derived peptide hormone that plays a major role in the stimulation of bone marrow stem cells and erythropoiesis [5–7]. EPO can promote retinal angiogenesis independently of VEGF in proliferative diabetic retinopathy [8, 9]. In diabetic retinas, EPO protects retinal cells by upregulating ZnT8 via ERK pathway activation and HIF-1α expression inhibition [10], and EPO may slow progression of diabetic nephropathy (DN) [11, 12]. EPO can also improve cardiac function by suppressing endoplasmic reticulum stress and inducing SERCA2a expression [13].

Several studies have examined associations between EPO polymorphisms and diabetic complications; however, the results have been inconsistent. Fan, *et al.* suggested that erythropoietin polymorphisms increased diabetic retinopathy risk [14]. Conversely, Balasubbu, *et al.* found no significant association [15]. Thus, our study investigated relationships between single-nucleotide polymorphisms (SNPs) in the EPO gene and diabetic complications.

## RESULTS

### Study characteristics

**Figure 1:**
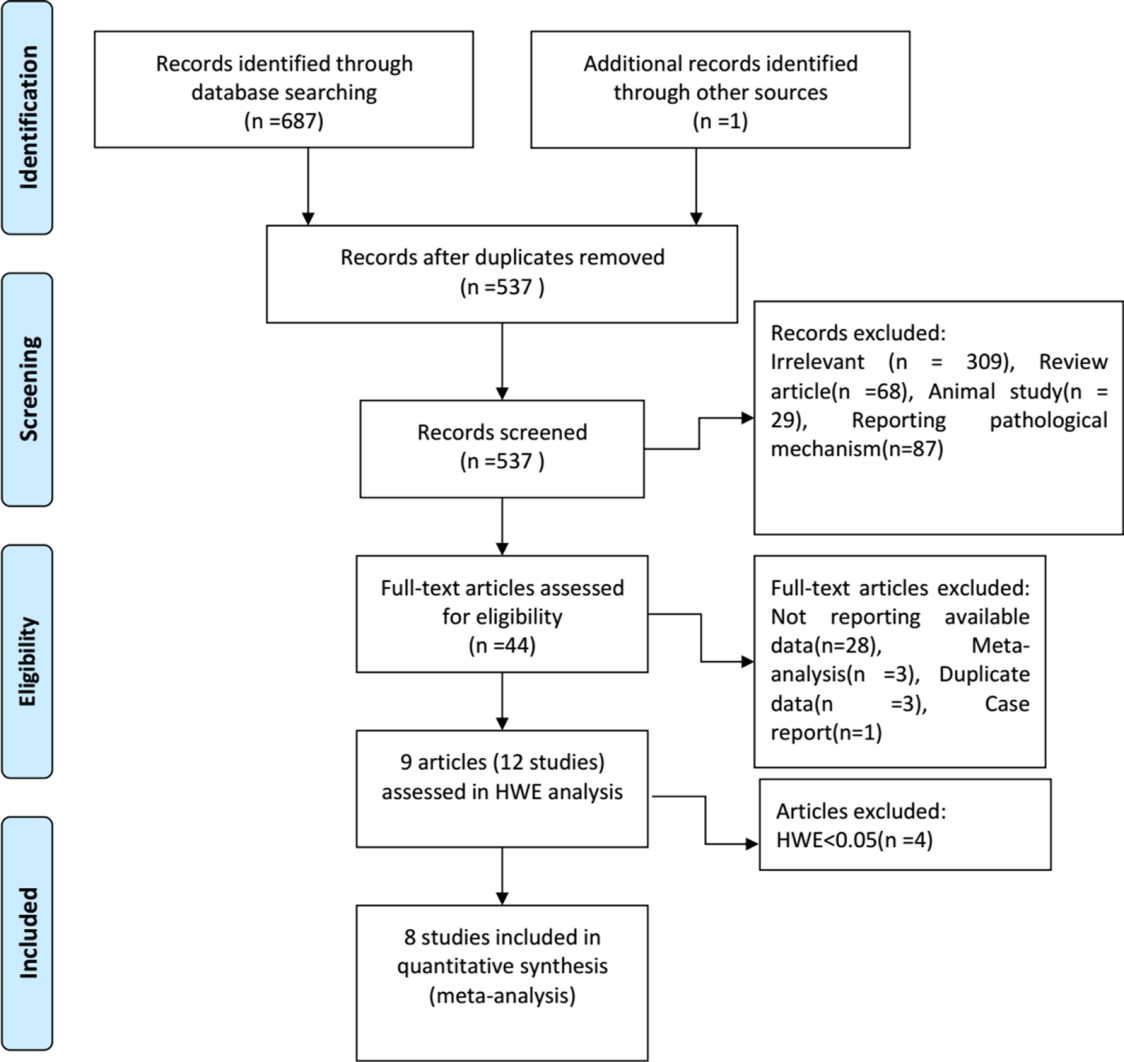
Study selection flow chart

Figure 1 shows our study selection process. In total, 688 studies were retrieved from the PubMed, Embase, Cochrane library, Web of Science, Wanfang, and Chinese National Knowledge Infrastructure databases. Of these, 151 duplicates were excluded from this study. Another 493 studies were excluded after reviewing the titles and abstracts. 44 eligible studies were evaluated for full-text review. Of these, 35 were excluded due to non-reporting of available data, duplicate data, or because they were meta-analyses and case reports. Nine original articles containing 12 studies remained after full-text review (Tables 1–2) [14–22].

**Table 1:** Main characteristics of studies included in the meta-analysis, by EPO SNP

SNP	Diabetic microvascular complications	Author	Year	Region	Type of diabetes mellitus	Genotyping method	Case	Control	Case					Control					HWE	Quality
									TT	TG	GG	T	G	TT	TG	GG	T	G		
rs1617640	Diabetic retinopathy	Abhary, *et al.* (1) (T1DM)	2010	Australia	T1DM	Sequencing	106	67	40	44	18	124	80	24	30	11	78	52	0.76	6
		Abhary, *et al.* (2) (T2DM)	2010	Australia	T2DM	Sequencing	179	166	65	78	27	208	132	64	88	11	216	110	0.01	6
		Balasubbu, *et al.*	2010	Indian	T2DM	Taqman assay	345	359	32	163	150	227	463	30	171	158	231	487	0.08	6
		Yang, *et al.*	2014	China	T2DM	Sequencing	216	284	146	55	10	347	75	182	82	16	446	114	0.11	7
		Zhang	2014	China	T2DM	PCR-LDR	448	344	293	138	13	724	164	225	98	15	548	128	0.31	6
		Gong	2015	China	T2DM	Mass spectrometry	128	128	77	40	11	194	62	92	36	0	220	36	0.06	6
		Li, et al.	2016	China	T2DM	PCR	191	130	58	121	71	237	263	27	51	9	105	69	0.04	7
		Fan, et al.	2016	China	T2DM	Taqman assay	397	796	208	161	28	577	217	468	302	26	1238	354	0.01	6
	Diabetic retinopathy and end- stage renal disease	Tong, *et al.* (1)	2008	America	T2DM	SNaPshot	374	239	150	172	52	472	276	66	127	46	259	219	0.28	7
		Tong, *et al.* (2)	2008	America	T1DM	SNaPshot	865	574	335	419	111	1089	641	148	307	119	603	545	0.08	7
		Tong, *et al.* (3)	2008	America	T1DM	SNaPshot	379	141	139	180	60	458	300	35	78	28	148	134	0.20	7
	Diabetic nephropathy	Alwohhaib, *et al.*	2014	Kuwait	T2DM	Taqman assay	76	128	NA	NA	NA	73	3	NA	NA	NA	118	10	NA	4
									TT	TC	CC	T	C	TT	TC	CC	T	C		
rs507392	Diabetic retinopathy	Abhary, *et al.* (1) (T1DM)	2010	Australia	T1DM	Sequencing	106	67	40	44	18	124	80	24	30	11	78	52	0.76	6
		Abhary, *et al.* (2) (T2DM)	2010	Australia	T2DM	Sequencing	179	166	65	78	27	208	132	63	88	11	214	110	0.01	6
		Zhang	2014	China	T2DM	PCR-LDR	448	344	281	149	14	711	177	225	98	24	548	146	0.01	6
		Fan, et al.	2016	China	T2DM	Taqman assay	397	796	202	161	34	565	229	463	305	28	1231	361	0.01	6
									AA	AC	CC	A	C	AA	AC	CC	A	C		
rs551238	Diabetic retinopathy	Abhary, *et al.* (1) (T1DM)	2010	Australia	T1DM	Sequencing	106	67	40	44	18	124	80	24	30	11	78	52	0.76	6
		Abhary, *et al.* (2) (T2DM)	2010	Australia	T2DM	Sequencing	179	166	65	78	27	208	132	64	88	11	216	110	0.01	6
		Zhang	2014	China	T2DM	PCR-LDR	448	344	286	140	13	712	166	219	92	24	530	140	0.002	6
		Yang, *et al.*	2014	China	T2DM	Sequencing	216	284	141	65	10	347	85	182	79	17	443	113	0.04	7
		Gong	2015	China	T2DM	Mass spectrometry	128	128	76	40	12	192	64	90	36	2	216	40	0.45	6
		Fan, et al.	2016	China	T2DM	Taqman assay	397	796	203	156	38	562	232	452	299	45	1203	389	0.63	6

SNP, single-nucleotide polymorphism; PCR-LDR, polymerase chain reaction-ligation detection reaction; T1DM, type 1 diabetes mellitus; T2DM, type 2 diabetes mellitus; NA, not available; HWE, Hardy-Weinberg equilibrium

**Table 2:** Main characteristics of studies included in the meta-analysis, by individual study

Variables	Abhary (2010)	Balasubbu (2010)	Yang (2014)	Zhang (2014)	Gong (2015)	Li (2016)	Fan (2016)	Tong (2008)	Alwohhaib (2014)
Ethnicity	Australian	Asian	Asian	Asian	Asian	Asian	Asian	American	Asian
Mean age (SD) Case vs. Control	T1DM (*P* < 0.001) 48.6(16.0) Vs. 36.3(14.6) T2DM(*P* = 0.82) 64.2(11.1) vs. 64.5(15.1)	57(9) vs. 59(11) *P* = 0.37	50.67(9.41) vs. 52.7(7.61) *P* = 0.008	62.35(11.92) vs. 60.16(11.67) *P* = 0.13	62.19(8.26) vs. 67.17(9.95) *P* = 0.019	63.10(6.83) vs. 68.48(6.91) *P* < 0.001	56.1(14.2) vs. 56.9(14.1) *P* = 0.357	68 vs. 70 67 vs. 44 40 vs. 52	NA
Males(%), mean(SD) Case vs. Control	T1DM (*P* = 0.87) 54(51) vs.35(52) T2DM (*P* < 0.001) 114 (63) vs. 73 (44)	242 (70) vs. 208 (58) *P* = 0.05	103 (48) vs.111 (39) *P* = 0.054	196 (44) vs. 163 (47) *P* = 0.31	61 (48) vs. 58 (45) *P* = 0.707	98 (51) vs.73 (56) *P* = 0.517	187 (47.1) vs. 392 (49.2) *P* = 0.534	232 (62) vs. 150 (63) 437 (51) vs. 247 (49) 230 (40) vs. 191 (50)	NA
Duration of DM (years) mean (SD) Case vs. Control	T1DM (*P* < 0.001) 28.1 (11.7) vs.12.43 (8.1) T2DM (*P* < 0.001) 17.3 (8.4) vs. 12.6 (8.9)	14 (9) Vs. 14 (9) *P* = 0.42	13.44 (7.22) vs.14.79 (4.97) *P* = 0.013	13.61 (7.31) vs. 10.70 (7.01) *P* = 0.001	14.27 (6.70) vs. 8.07 (5.44) *P* < 0.001	15.74 (7.48) vs. 17.74 (7.10) *P* < 0.001	9.5 (3.8) vs. 5.7 (3.0) *P* < 0.001	22 vs. 19 49 vs. 32 25 vs. 35	NA
Smoker (%), mean (SD) Case vs. Control	T1DM (*P* = 0.93) 54 (51) vs. 34 (51) T2DM (*P* = 0.52) 99 (55) vs. 86 (52)	NA	NA	NA	NA	NA	148 (37.3) vs. 273 (34.2) *P* = 0.321	NA	NA
HbA1c level, % mean (SD) Case vs. Control	T1DM (*P* = 0.07) 9.2 (7.5) vs. 7.5 (2.0) T2DM (*P* = 0.005) 8.5 (8.6) vs. 6.5 (3.1)	6.4 (1.6) vs. 6.4 (1.2) *P* = 0.09	7.84 (1.70) vs.6.97 (1.40) *P* < 0.001	9.31 (2.88) vs. 8.57 (2.03) *P* = 0.07	8.06 (1.54) vs. 7.74 (1.91) *P* = 0.426	7.45 (0.49) vs. 6.84 (0.85) *P* < 0.001	NA	8 vs. 7.6 10.9 vs. 7.8 7.4 vs. 8.1	NA
BMI, mean (SD) Case vs. Control	T1DM (*P* = 0.73) 25.4 (10.1) vs. 25.9 (6.7) T2DM (*P* = 0.01) 29.7 (10.9) vs. 32.5 (9.1)	NA	25.8 (4.13) vs. 25.26 (3.95) *P* = 0.14	25.58 (4.18) vs. 26.16 (4.75) *P* = 0.30	23.84 (2.71) vs. 23.71 (2.82) *P* = 0.842	NA	23.4 (6.7) vs. 25.3 (6.2) *P* < 0.001	NA	NA

NA, not available; T1DM, type 1 diabetes mellitus; T2DM, type 2 diabetes mellitus; SD, standard deviation.

Hardy-Weinberg equilibrium (HWE) was assessed via Chi-square test in the control subjects of each study. Genotype distributions among controls were consistent with HWE in only eight studies [15–20]. These eight studies, which investigated three SNPs (rs1617640, rs507392 and rs551238), were included in the final meta-analysis. The eight studies included 2,861 cases and 2,136 controls. Five studies focused on diabetic retinopathy, and three focused on diabetic retinopathy and end-stage renal disease. Four examined patients with type 2 diabetes mellitus (T2DM), and one examined patients with type 1 diabetes mellitus (T1DM). Four included patients of Asian descent, three included patients of American descent, and one included patients of Australian descent. According to the Newcastle-Ottawa quality assessment scale (NOS), quality scores ranged from 6–7, and the median score of the case-control studies was 6.5.

### EPO rs1617640 quantitative analyses

**Figure 2:**
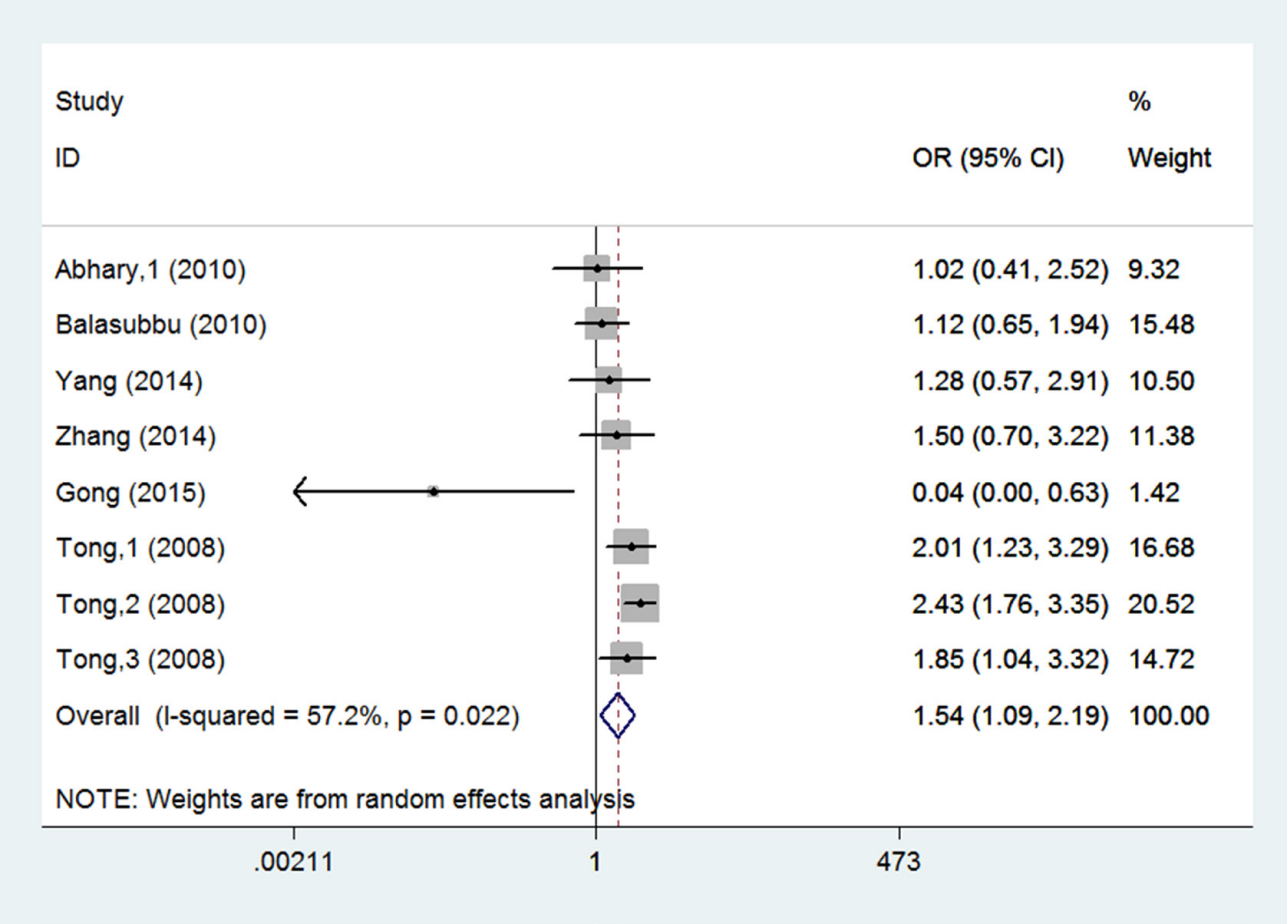
Forest plots of the association between rs1617640 polymorphisms and diabetic microvascular complication susceptibility. (TT vs. GG) Squares and horizontal lines correspond to the study-specific OR and 95% CI. The diamond represents the summary OR and 95% CI.

**Figure 3:**
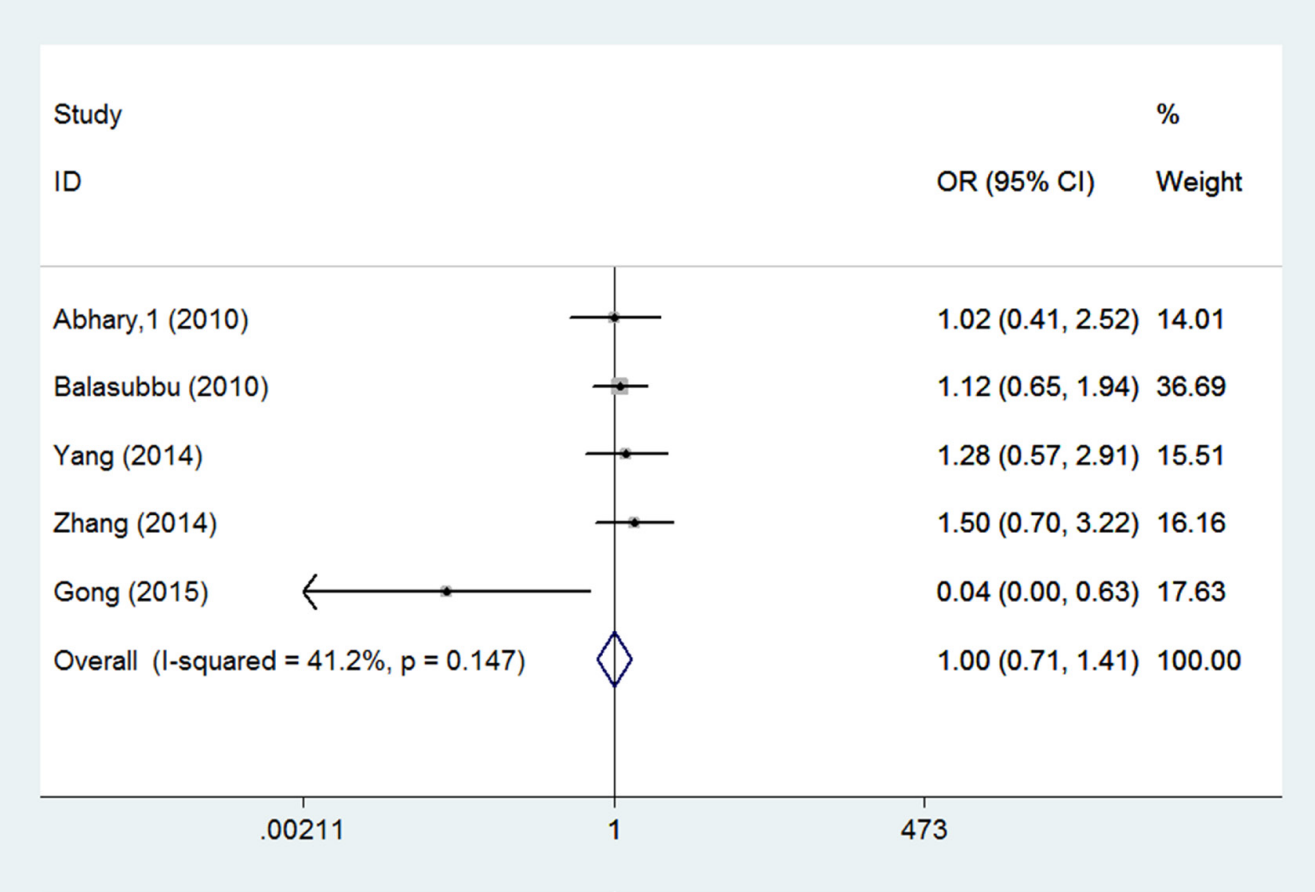
Forest plots of the association between rs1617640 polymorphisms and diabetic retinopathy susceptibility (TT vs. GG) Squares and horizontal lines correspond to the study-specific OR and 95% CI. The diamond represents the summary OR and 95% CI.

Eight studies investigated the association between EPO rs1617640 gene polymorphisms and diabetic complications risk [15–20]. Differences were observed in the genotype model (TT vs. GG: OR = 1.544, 95% CI = 1.089–2.189, *P* = 0.015) (Figure 2, Table 3). For diabetic retinopathy, rs1617640 polymorphisms were not associated with increased complication risk in any genetic model (allele model: OR = 0.960, 95% CI = 0.769–1.198, *P* = 0.717; genotype model: TG vs. GG: OR = 0.975, 95% CI = 0.756–1.259, *P* = 0.847, TT vs. GG: OR = 1.003, 95% CI = 0.714–1.410, *P* = 0.985; dominant model: OR = 0.988, 95% CI = 0.774–1.261, *P* = 0.923; recessive model: OR = 0.990, 95% CI = 0.820–1.196, *P* = 0.921) (Figure 3). In T2DM and Asian populations, no association was observed in any genetic model (allele model: OR = 0.940, 95% CI = 0.721–1.227, *P* = 0.651; genotype model: TG vs. GG: OR = 0.983, 95% CI = 0.753–1.283, *P* = 0.898, TT vs. GG: OR = 1.075, 95% CI = 0.552–2.093, *P* = 0.832; dominant model: OR = 1.057, 95% CI = 0.601–1.859, *P* = 0.846; recessive model: OR = 0.980, 95% CI = 0.804–1.195, *P* = 0.844). For diabetic retinopathy and end-stage renal disease, rs1617640 polymorphisms were associated with diabetic microvascular complications in all genetic models (allele model: OR = 1.486, 95% CI = 1.324–1.667, *P* = 0.000; genotype model: TG vs. GG: OR = 1.317, 95% CI = 1.052–1.648, *P* = 0.016, TT vs. GG: OR = 2.209, 95% CI = 1.730–2.821, *P* = 0.000; dominant model: OR = 1.607, 95% CI = 1.298–1.990, *P* = 0.000; recessive model: OR = 1.792, 95% CI = 1.502–2.138, *P* = 0.000). Only one study assessed T1DM in Australian populations, and no association was found in any genetic model (allele model: OR = 1.033, 95% CI = 0.659–1.620, *P* = 0.886; genotype model: TG vs. GG: OR = 0.896, 95% CI = 0.371–2.165, *P* = 0.808, TT vs. GG: OR = 1.019, 95% CI = 0.412–2.517, *P* = 0.968; dominant model: OR = 0.951, 95% CI = 0.417–2.168, *P* = 0.904; recessive model: OR = 1.102, 95% CI = 0.580–2.094, *P* = 0.766).

**Table 3:** ORs and 95% CIs for EPO gene polymorphisms and diabetic microvascular complications using different genetic models

SNP	Comparison	Subgroup	N	Heterogeneity test		Z test P_Z_	Publication bias		OR and 95% CI	
				P_H_	I^2^ (%)		P_B_	P_E_	Fixed model	Random model
rs1617640	T vs. G	Overall	8	0.000	76.4	0.180	0.174	0.037	1.251 (1.146, 1.365)	1.142 (0.940, 1.387)
		DR	5	0.049	58.1	0.717	0.221	0.356	0.991 (0.867, 1.134)	0.960 (0.769, 1.198)
		T2DM	4	0.023	68.5	0.651			0.987 (0.857, 1.137)	0.940 (0.721, 1.227)
		T1DM	1			0.886			1.033 (0.659, 1.620)	1.033 (0.659, 1.620)
		Asian	4	0.023	68.5	0.651			0.987 (0.857, 1.137)	0.940 (0.721, 1.227)
		Australian	1			0.886			1.033 (0.659, 1.620)	1.033 (0.659, 1.620)
		DR+DN	3	0.780	0	0.000			1.486 (1.324, 1.667)	1.485 (1.324, 1.667)
	TG vs. GG	Overall	8	0.246	23.0	0.095	0.174	0.163	1.154 (0.975, 1.366)	1.169 (0.944, 1.447)
		DR	5	0.202	32.9	0.847	0.221	0.462	0.975 (0.756, 1.259)	1.028 (0.679, 1.555)
		T2DM	4	0.118	48.9	0.898			0.983 (0.753, 1.283)	1.040 (0.597, 1.810)
		T1DM	1			0.808			0.896 (0.371, 2.165)	0.896 (0.371, 2.165)
		Asian	4	0.118	48.9	0.898			0.983 (0.753, 1.283)	1.040 (0.597, 1.810)
		Australian	1			0.808			0.896 (0.371, 2.165)	0.896 (0.371, 2.165)
		DR+DN	3	0.544	0	0.016			1.317 (1.052, 1.648)	1.317 (1.051, 1.649)
	TT vs. GG	Overall	8	0.022	57.2	0.015	0.019	0.002	1.677 (1.377, 2.043)	1.544 (1.089, 2.189)
		DR	5	0.147	41.2	0.985	0.221	0.092	1.003 (0.714, 1.410)	1.098 (0.663, 1.820)
		T2DM	4	0.078	56.0	0.832			1.001 (0.693, 1.444)	1.075 (0.552, 2.093)
		T1DM	1			0.968			1.019 (0.412, 2.517)	1.019 (0.412, 2.517)
		Asian	4	0.078	56.0	0.832			1.001 (0.693, 1.444)	1.075 (0.552, 2.093)
		Australian	1			0.968			1.019 (0.412, 2.517)	1.019 (0.412, 2.517)
		DR+DN	3	0.665	0	0.000			2.209 (1.730, 2.821)	2.208 (1.729, 2.820)
	TT+TG vs. GG	Overall	8	0.050	50.3	0.059	0.063	0.185	1.296 (1.104, 1.522)	1.294 (0.991, 1.691)
		DR	5	0.162	38.9	0.923	0.462	0.463	0.988 (0.774, 1.261)	1.054 (0.692, 1.606)
		T2DM	4	0.090	53.9	0.846			0.992 (0.768, 1.280)	1.057 (0.601, 1.859)
		T1DM	1			0.904			0.951 (0.417, 2.168)	0.951 (0.417, 2.168)
		Asian	4	0.090	53.9	0.846			0.992 (0.768, 1.280)	1.057 (0.601, 1.859)
		Australian	1			0.904			0.951 (0.417, 2.168)	0.951 (0.417, 2.168)
		DR+DN	3	0.538	0	0.000			1.607 (1.298, 1.990)	1.607 (1.297, 1.991)
	TT vs. TG+GG	Overall	8	0.001	72.2	0.087	0.019	0.002	1.365 (1.201, 1.551)	1.257 (0.968, 1.632)
		DR	5	0.274	22.1	0.921	0.221	0.092	0.990 (0.820, 1.196)	0.986 (0.789, 1.233)
		T2DM	4	0.170	40.2	0.844			0.980 (0.804, 1.195)	0.968 (0.740, 1.266)
		T1DM	1			0.766			1.102 (0.580, 2.094)	1.102 (0.580, 2.094)
		Asian	4	0.170	40.2	0.844			0.980 (0.804, 1.195)	0.968 (0.740, 1.266)
		Australian	1			0.766			1.102 (0.580, 2.094)	1.102 (0.580, 2.094)
		DR+DN	3	0.981	0	0.000			1.792 (1.502, 2.138)	1.792 (1.502, 2.138)
rs507392	T vs. C	Overall	1			0.886			1.033 (0.659, 1.620)	1.033 (0.659, 1.620)
	TC vs. CC	Overall	1			0.808			0.896 (0.371, 2.165)	0.896 (0.371, 2.165)
	TT vs. CC	Overall	1			0.968			1.019 (0.412, 2.517)	1.019 (0.412, 2.517)
	TT+TC vs. CC	Overall	1			0.904			0.951 (0.417, 2.168)	0.951 (0.417, 2.168)
	TT vs. TC+CC	Overall	1			0.766			1.102 (0.580, 2.094)	1.102 (0.580, 2.094)
rs551238	A vs. C	Overall	3	0.151	47.1	0.002			0.774 (0.658, 0.911)	0.769 (0.585, 1.012)
	AC vs. CC	Overall	3	0.225	33.0	0.011			0.598 (0.402, 0.890)	0.596 (0.331, 1.073)
	AA vs. CC	Overall	3	0.086	59.2	0.104			0.529 (0.358, 0.782)	0.515 (0.231, 1.147)
	AA+AC vs. CC	Overall	3	0.112	54.3	0.003			0.561 (0.385, 0.817)	0.546 (0.268, 1.111)
	AA vs. AC+CC	Overall	3	0.384	0	0.026			0.791 (0.643, 0.973)	0.791 (0.643, 0.973)

*P*_H_: P-value of heterogeneity test; *P*_Z_: P-value of Z test; *P*_B_: P-value of Begg's test; *P*_E_: P-value of Egger's test; OR: odds ratio; 95% CI: 95% confidence interval; N: number of comparisons

### EPO rs507392 quantitative analyses

Only one study investigated the relationship between EPO rs507392 polymorphisms and diabetic retinopathy [16]. No association was observed in any genetic model (allele model: OR = 1.033, 95% CI = 0.659–1.620, *P* = 0.886; genotype model: TC vs. CC: OR = 0.896, 95% CI = 0.371–2.165, *P* = 0.808, TT vs. CC: OR = 1.019, 95% CI = 0.412–2.517, *P* = 0.968; dominant model: OR = 0.951, 95% CI = 0.417–2.168, *P* = 0.904; recessive model: OR = 1.102, 95% CI = 0.580–2.094, *P* = 0.766.

### EPO rs551238 quantitative analyses

**Figure 4:**
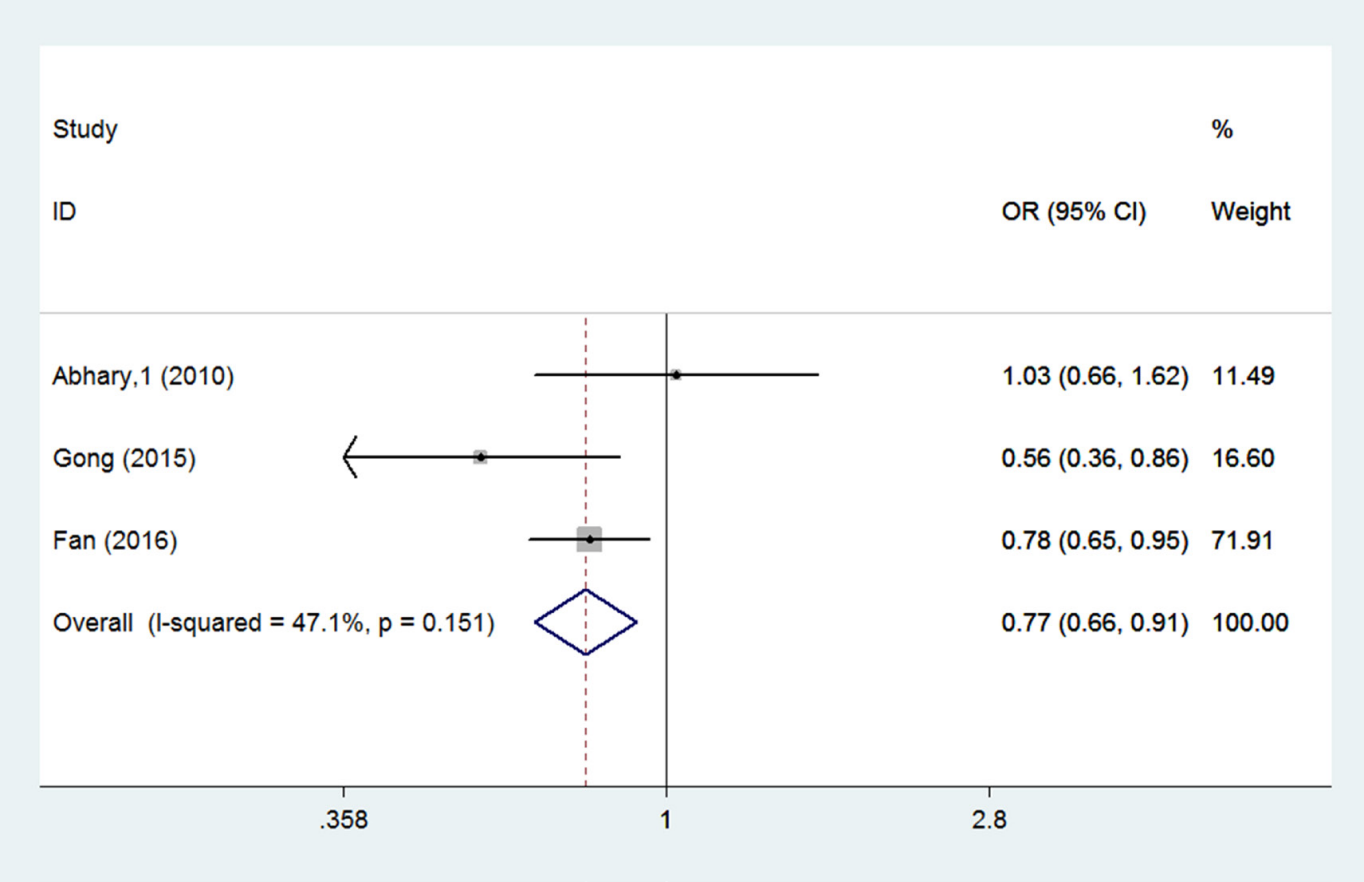
Forest plots of the association between rs551238 polymorphisms and diabetic retinopathy susceptibility (A vs C). Squares and horizontal lines correspond to the study-specific OR and 95% CI. The diamond represents the summary OR and 95% CI.

Three studies were eligible for the EPO rs551238 meta-analysis [14, 16, 20]. An association was found between EPO rs551238 polymorphisms and diabetic retinopathy (allele model: OR = 0.774, 95% CI = 0.658–0.911, *P* = 0.002; genotype model: AC vs. CC: OR = 0.598, 95% CI = 0.402–0.890, *P* = 0.011; dominant model: OR = 0.561, 95% CI = 0.385–0.817, *P* = 0.003; recessive model: OR = 0.791, 95% CI = 0.643–0.973, *P* = 0.026) (Figure 4).

### Publication bias and sensitivity analysis

**Figure 5:**
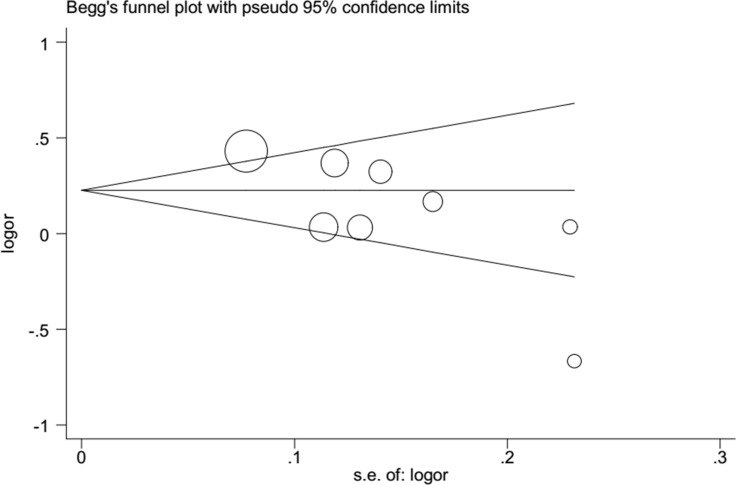
Publication bias tested by Begg's funnel plot (rs1617640 T vs G). Each point represents a separate study for the indicated association. Logor, natural logarithm of OR. Horizontal line, mean effect size.

**Figure 6:**
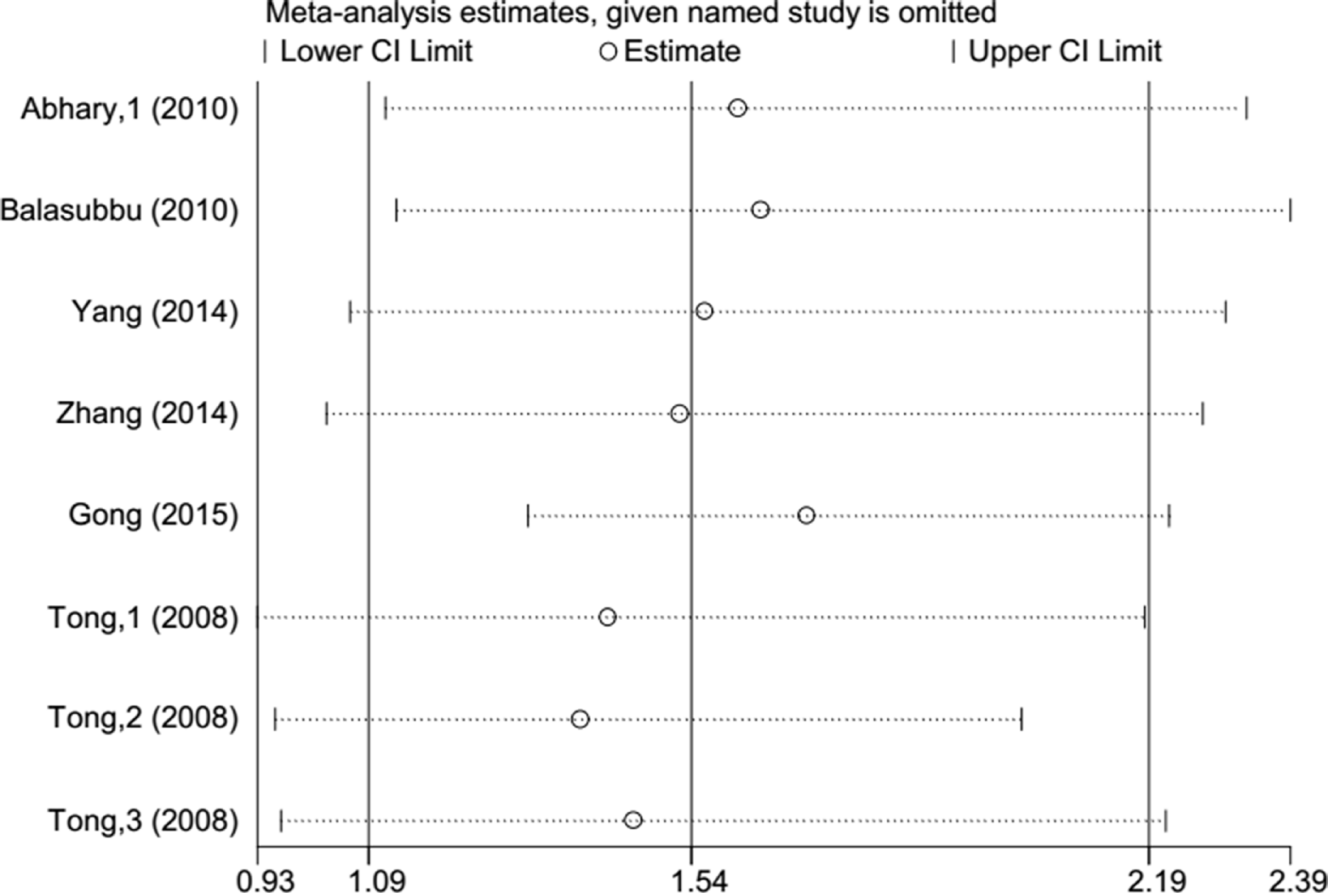
Sensitivity analysis of each study performed by omitting each data set from the analysis (rs1617640 TT vs GG).

Begg's funnel plot and Egger's test were employed to detect publication bias in this meta-analysis. We found no evidence of publication bias, with the exception of rs1617640 (TT vs. GG, TT vs. TG+GG), which exhibited a slight publication bias (Figure 5, Table 3). A sensitivity analysis was performed to examine the influence of excluding each study on the pooled ORs. The overall effects were not altered when each study was omitted, suggesting that our meta-analysis results were reliable (Figure 6).

## DISCUSSION

EPO is located on chromosome 7q21 and encodes a potent angiogenic factor expressed in the retina and kidney [23–25]. Several studies have assessed the impacts of EPO single-nucleotide polymorphisms (SNPs) on diabetic microvascular complications; however, the results have been inconsistent. We performed this meta-analysis to obtain conclusive results regarding the relationship between EPO polymorphisms and diabetic microvascular complications. We evaluated the most commonly investigated EPO polymorphisms: rs1617640, rs507392, and rs551238. Our results indicate that EPO polymorphisms are a risk factor for diabetic microvascular complications.

We searched several databases for relevant studies assessing the association between the EPO polymorphisms and diabetic complications. Our analysis included eight studies with a combined total of 2,861 cases and 2,136 controls. These studies were conducted between 2008 and 2015 to investigate the association between EPO polymorphisms and diabetic retinopathy and nephropathy. Tong, *et al.* suggested an association between the T allele of SNP rs1617640 and proliferative diabetic retinopathy as well as end-stage renal disease [19]. Gong associated higher diabetic retinopathy risk with carrying the TT/GT genotype and T allele at the EPO gene site rs1617640 [20]. Abhary, *et al.* concluded that all three EPO SNPs (rs507392, rs1617640, and rs551238) were associated with diabetic retinopathy risk independent of duration of DM, degree of glycemic control, and nephropathy [16]. However, some studies found no associations [15]. In our study, the pooled results indicated a relationship between rs1617640 polymorphisms and diabetic complications (genotype model: TT vs. GG: OR = 1.544, 95% CI = 1.089–2.189, *P* = 0.015).

Our subgroup study stratified by complication type showed that rs1617640 polymorphisms were associated with diabetic retinopathy and end-stage renal disease. However, the association with diabetic retinopathy was not significant in any model. Only one study evaluated T1DM in Australian populations, so we were not able to evaluate statistical significance in this case.

Only one study investigated rs507392 polymorphisms, and found no association between these polymorphisms and diabetic retinopathy. Three studies investigated the association between EPO rs551238 polymorphisms and diabetic retinopathy risk, with inconsistent results. We pooled the results and found a relationship between rs551238 polymorphisms and diabetic retinopathy.

Our meta-analysis had some limitations. First, the number of included studies was relatively small. rs1617640 polymorphisms were examined in eight studies, and rs507392 and rs551238 polymorphisms were investigated in one and three studies, respectively. Previous studies demonstrated that EPO variations played roles in DR and DN development [19, 26], and different results were found for T2DM and T1DM. Thus, we performed a subgroup study stratified by complication type, DM type, and ethnicity. However, some of our stratified analyses were limited by small sample sizes. Three studies focused on DR and DN. Only one study assessed T1DM in an Australian population. There were some differences between cases and controls, including patient age, gender, duration of DM, HbA1c level and BMI (Table 2). Stratification analyses based on differences in DM duration, gender, or other factors could not be performed because of limited sample sizes. Data from large, multi-center studies, including DM severity, ethnicity, and other complications and factors, are needed to confirm these relationships. Second, due to the limited number of studies, we did not investigate publication bias for the rs551238 polymorphism. A small publication bias was detected for the rs1617640 polymorphism in some models (TT vs. GG, TT vs. TG + GG), indicating that some unpublished studies might have been overlooked. Third, heterogeneity was found between the rs1617640 polymorphism studies; however, we could not conduct subgroup analyses to investigate the heterogeneity source due to the limited number of studies. Finally, we did not examine other risk factors, such as environmental effects and genetic factors.

In summary, we found that EPO polymorphisms are a risk factor for diabetic microvascular complications. rs1617640 polymorphisms are associated with increased risk of diabetic retinopathy and nephropathy, and rs551238 polymorphisms are associated with increased risk of diabetic retinopathy.

## MATERIALS AND METHODS

### Search for eligible studies

Relevant studies were retrieved from the PubMed, Embase, Cochrane library, Web of Science, Wanfang, and Chinese National Knowledge Infrastructure databases. The last search was performed on August 22, 2017, with keywords including (“erythropoietin” OR “erythropoietin-generating factor” OR “renal erythropoietic factor” OR “erythropoitin-generating factor” OR “EPO” OR “rs1617640” OR “rs507392” OR “rs551238”) AND (“gene” OR “Polymorphisms, Genetic” OR “Polymorphisms, Genetic” OR “Genetic Polymorphism” OR “Polymorphism (Genetics)” OR “Genetic Polymorphisms” OR “Polymorphism” OR “genetic” OR “allele” OR “variation” OR “variant” OR “mutation”) AND (“Diabetes” OR “Diabetes-Related ” OR “Diabetic ”) AND (“Complication” OR “Complications” OR “Disease, Retinal” OR” Diseases, Retinal “ OR “Retinal Disease” OR “retinopathy” OR “nephropathy” OR “ Disease, Kidney” OR “ Kidney Disease” OR “vascular” OR “Blood Vessel” OR “Vessel, Blood” OR “Vessels, Blood” OR “Neuropathy” OR “Neuropathies” OR “Neuralgia” OR “Neuralgias” OR “Cardiomyopathy” OR “Myocardial Diseases” OR “Disease, Myocardial” OR “Diseases, Myocardial” OR “Myocardial Disease” OR “Myocardiopathies” OR “Myocardiopathy”). We also manually searched the reference lists of relevant reports to identify additional studies.

### Inclusion and exclusion criteria

All selected studies in this analysis met the following criteria: (1) case-control studies; (2) explored the correlation between EPO polymorphisms and diabetic microvascular complications; and (3) contained sufficient data for calculating odds ratios (ORs) and 95% confidence intervals (95% CIs). Exclusion criteria were as follows: (1) studies without Hardy-Weinberg equilibrium (HWE) in the control groups; (2) studies not relevant to diabetic complications or lacking a control population; and (3) studies lacking sufficient data for quantitative analyses.

### Data extraction and quality assessment

Two investigators (Li H and Xu HP) independently reviewed all articles and extracted available data from individual studies. Any discrepancies were resolved by discussion between these two investigators. The following information was extracted from each eligible study: (1) first author, (2) year of publication, (3) country of origin and ethnicity of study participants, (5) type of diabetes mellitus and complications, (6) genotyping method, (7) number of cases and controls, (8) polymorphism and genotype distribution, and HWE for controls. We assessed the quality of each eligible study according to the Newcastle-Ottawa quality assessment scale (NOS) [27]. Quality score ranged from 0–9.

### Statistical analysis

HWE was assessed via Chi-square test in the control populations of each study. Pooled ORs and corresponding 95% CIs were calculated to estimate associations between EPO polymorphisms and diabetic microvascular complications. Z test was used to assess the overall effect and *P* < 0.05 was considered statistically significant. The strength of the association was determined using the following models: allele model (T vs. G, T vs. C, A vs. C), genotype model (TG vs. GG, TT vs. GG, TC vs. CC, TT vs. CC, AC vs. CC, AA vs. CC), dominant model (TT/TG vs. GG, TT/TC vs. CC, AA/AC vs. CC), and recessive model (TT vs. TG/GG, TT vs. TC/CC, AA vs. AC/CC). Subgroup analyses were conducted according to ethnicity, type of diabetes mellitus, and complication. We used the *Q*-statistic and I^2^ statistic to evaluate statistical heterogeneity among studies [27]. *P* ≥ 0.1 and I^2^ < 50% suggested a lack of heterogeneity among studies. A random-effect model was used through the DerSimonian and Laird method in the presence of heterogeneity (*P* < 0.1 or I^2^ > 50%); otherwise, a fixed-effect model based on the Mantel-Haenszel method was employed (*P* ≥ 0.1 or I^2^ < 50%). We conducted a sensitivity analysis to assess the stability of the results. Egger's test and Begg's funnel plot were used to evaluate publication bias. Analyses were performed using STATA version 11.0 software (Stata Corporation, College Station, TX, USA).
